# Crystal structure of {6,6′-dihy­droxy-2,2′-[imino­bis­(propane-1,3-diyl­nitrilo­methanylyl­idene)]diphenolato-κ^5^ *O* ^1^,*N*,*N*′,*N*′′,*O* ^1′^}copper(II)

**DOI:** 10.1107/S2056989015019684

**Published:** 2015-10-24

**Authors:** Shabana Noor, Sarvendra Kumar, Suhail Sabir, Rüdiger W. Seidel, Richard Goddard

**Affiliations:** a Department of Chemistry, Aligarh Muslim University, Aligarh 202 002, India; b Faculty of Pharmaceutical Science, Tokyo University of Science, Noda, Japan; c Max-Planck-Institut für Kohlenforschung, Kaiser-Wilhelm-Platz-1, 45470 Mülheim an der Ruhr, Germany

**Keywords:** crystal structure, Cu^II^ complex, Schiff bases, distorted trigonal bipyramidal coordination geometry, hydrogen bond

## Abstract

The title compound, [Cu(C_20_H_23_N_3_O_4_)], crystallizes in the space group *Cc* with two independent mol­ecules in the asymmetric unit. The Cu^II^ atoms are each coordinated by the penta­dentate Schiff base ligand in a distorted trigonal bipyramidal N_3_O_2_ geometry. The equatorial plane is formed by the two phenolic O atoms and the amine N atom, while the axial positions are occupied by the two imine N atoms. In the crystal, the two independent mol­ecules are each connected into a column along the *b* axis through inter­molecular O—H⋯O hydrogen bonds. The two independent columns are further linked through an N—H⋯O hydrogen bond, forming a double-column structure.

## Related literature   

For characteristic properties of Schiff bases and their metal complexes, see: Averseng *et al.* (2001 ▸); Sanmartin *et al.* (2000 ▸); Brown & Wardeska (1982 ▸); Lan *et al.* (2009 ▸).
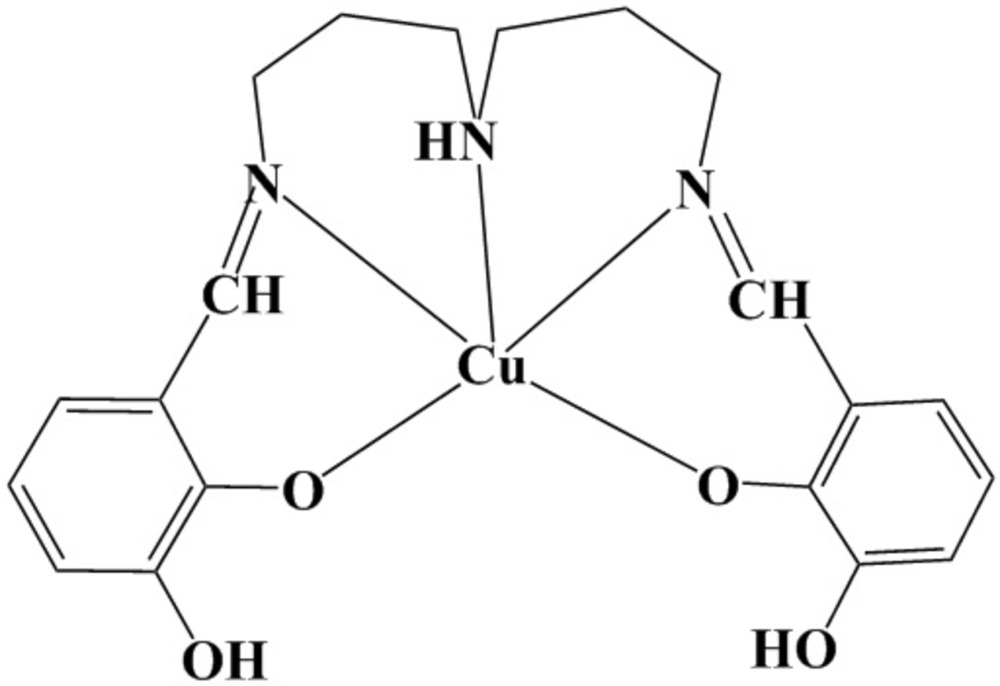


## Experimental   

### Crystal data  

[Cu(C_20_H_23_N_3_O_4_)]
*M* *_r_* = 432.95
Monoclinic 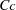

*a* = 28.4747 (8) Å
*b* = 6.186 (5) Å
*c* = 22.925 (12) Å
β = 114.792 (11)°
*V* = 3666 (3) Å^3^
*Z* = 8
Mo *K*α radiation
μ = 1.23 mm^−1^
*T* = 100 K
0.07 × 0.06 × 0.02 mm

### Data collection  

Enraf–Nonius KappaCCD diffractometer
Absorption correction: Gaussian (*SADABS*; Bruker, 2006 ▸) *T*_min_ = 0.904, *T*_max_ = 0.974
26717 measured reflections
12062 independent reflections
11048 reflections with *I* > 2σ(*I*)
*R*_int_ = 0.050

### Refinement  

*R*[*F* ^2^ > 2σ(*F* ^2^)] = 0.070
*wR*(*F* ^2^) = 0.182
*S* = 1.25
12062 reflections
510 parameters
8 restraints
H-atom parameters constrained
Δρ_max_ = 1.82 e Å^−3^
Δρ_min_ = −1.65 e Å^−3^
Absolute structure: Parsons & Flack (2004 ▸), 5103 Friedel pairs
Absolute structure parameter: 0.10 (2)

### 

Data collection: *DATCOL* (Bruker, 2006 ▸); cell refinement: *EVALCCD* (Duisenberg *et al.*, 2003 ▸); data reduction: *EVALCCD*; program(s) used to solve structure: *SHELXS97* (Sheldrick, 2008 ▸); program(s) used to refine structure: *SHELXL2014* (Sheldrick, 2015 ▸); molecular graphics: *DIAMOND* (Brandenburg, 1999 ▸); software used to prepare material for publication: *enCIFer* (Allen *et al.*, 2004 ▸).

## Supplementary Material

Crystal structure: contains datablock(s) I, New_Global_Publ_Block. DOI: 10.1107/S2056989015019684/is5426sup1.cif

Structure factors: contains datablock(s) I. DOI: 10.1107/S2056989015019684/is5426Isup2.hkl

**Fig. 1.**
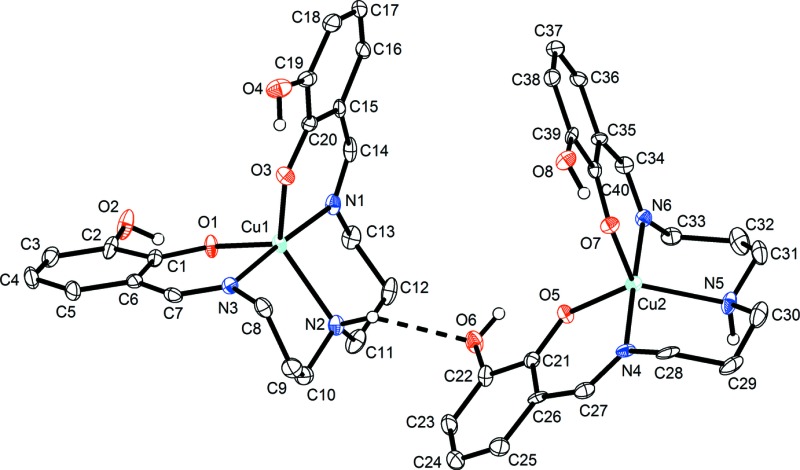
Asymmetric unit of the title compound. Displacement ellipsoids are drawn at the 50% probability level. H atoms are represented by small spheres of arbitrary radii. Carbon-bound H atoms are omitted for clarity. The intermolecular N—H···O hydrogen bond is illustrated by a dashed line.

. DOI: 10.1107/S2056989015019684/is5426fig1.tif
Asymmetric unit of the title compound. Displacement ellipsoids are drawn at the 50% probability level. H atoms are represented by small spheres of arbitrary radii. Carbon-bond H atoms are omitted for clarity. The dashed line illustrates a hydrogen bond

**Fig. 2.**
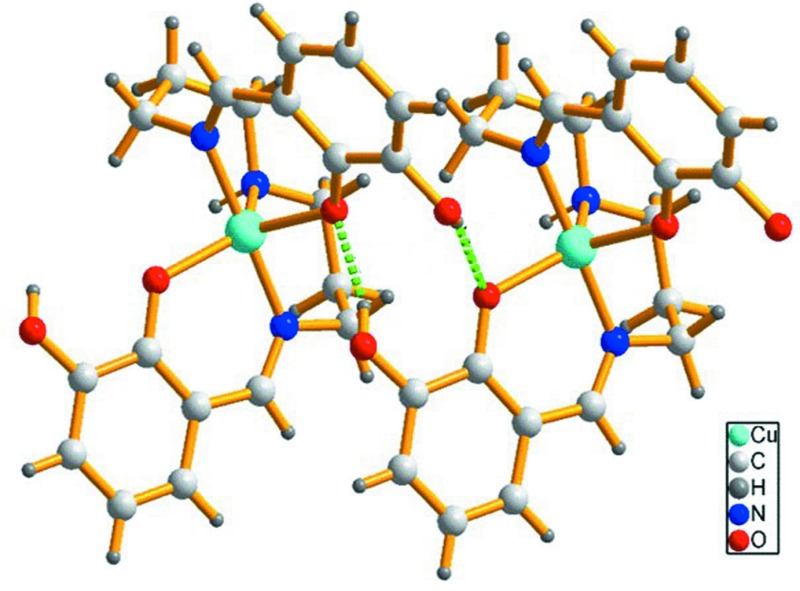
Intermolecular O—H···O hydrogen bonds between symmetry-related molecules along the crystallographic b axis.

. DOI: 10.1107/S2056989015019684/is5426fig2.tif
A hydrogen-bonded diagram of the title compound. The dashed lines show O—H⋯O hydrogen bonds.

CCDC reference: 1404639

Additional supporting information:  crystallographic information; 3D view; checkCIF report

## Figures and Tables

**Table 1:** Hydrogen-bond geometry (, )

*D*H*A*	*D*H	H*A*	*D* *A*	*D*H*A*
O2H2O3^i^	0.84	1.95	2.699(8)	147
O4H4*A*O1^ii^	0.84	1.90	2.686(8)	156
O6H6O7^i^	0.84	2.08	2.758(8)	137
O6H6O8^i^	0.84	2.40	3.101(8)	142
O8H8O5^ii^	0.84	2.05	2.806(7)	149
N2H2*A*O6	1.00	2.36	3.163(8)	137

Symmetry codes: (i) 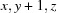; (ii) 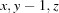.

## supplementary crystallographic information

### S1. Comment

Schiff bases and their coordination compounds play an important role in metal coordination chemistry. A large number of Schiff bases and their metal complexes have been synthesized and extensively studied because they have some characteristic properties such as manifestations of novel structures, thermal stability, relevant biological properties, high synthesis flexibility and medicinal utility (Brown & Wardeska, 1982; Averseng *et al.*, 2001; Sanmartin *et al.*, 2000; Lan *et al.*, 2009).

The title compound crystallizes in the non-centrosymmetric monoclinic space group *Cc* with two molecules in the asymmetric unit. Since the two independent molecules have a similar geometry, we restrict the following discussion to one molecule. The Schiff ligand coordinates the metal ions as a pentadentate [ONNNO] chelating ligand through the phenolic O atoms (O1 and O2) and one aminic (N2) and two iminic N atoms (N1 and N3). The equatorial plane of the trigonal bipyramidal geometry is formed by atoms N2, O1 and O3. The Cu1—N2 [2.226 (6) Å] bond is significantly longer than the other two Cu1—O1 [2.074 (5) Å] and Cu1—O3 [2.049 (5) Å]. The two axial bond lengths are almost equal [Cu1—N1 = 1.939 (6) and Cu1—N3 = 1.942 (6) Å]. Deviation from regular trigonal bipyramid is evident from the bond angles of N2—Cu1—O1 = 113.3 (2)°, N2—Cu1—O3 = 125.3 (2)° and O1—Cu1—O3 = 121.1 (2)°. The terminal OH groups of the ligands are uncoordinated.

### S2. Experimental

To a stirred solution of the Schiff base H_4_L (0.4 mmol, 0.148 mg) dissolved in (20 ml EtOH and 20 ml CH_3_CN) were added Cu(OAc)_2._H_2_O (0.4 mmol, 0.08 g) followed by Et_3_N (0.12 mmol, 0.16 ml). The resulting mixture was refluxed for 5–6 h. The reaction mixture was filtered. Dark colored crystals were obtained after 2–5 days by slow diffusion of diethyl ether into the solution.

### S3. Refinement

The structure was refined as an inversion twin. The anisotropic displacement parameters of atom C28 were restrained towards isotropy with a standard deviation of 0.005 Å. H atoms were placed at geometrically calculated positions with O—H = 0.84 Å, N—H = 1.00 Å, C(*sp*^2^)—H = 0.95 Å and C(*sp*^3^)—H = 0.99 Å and refined with a riding model. *U*_iso_(H) was set 1.2 times *U*_eq_(C, N) or 1.5 times *U*_eq_(O). The initial torsion angles of the hydroxy groups were determined *via* circular difference Fourier syntheses and subsequently refined while maintaining a tetrahedral angle at the O atom.

### Crystal data

**Table:**

[Cu(C_20_H_23_N_3_O_4_)]	*F*(000) = 1800
*M**_r_* = 432.95	*D*_x_ = 1.569 Mg m^−^^3^
Monoclinic, *C**c*	Mo *K*α radiation, λ = 0.71073 Å
*a* = 28.4747 (8) Å	Cell parameters from 32601 reflections
*b* = 6.186 (5) Å	θ = 2.9–33.1°
*c* = 22.925 (12) Å	µ = 1.23 mm^−^^1^
β = 114.792 (11)°	*T* = 100 K
*V* = 3666 (3) Å^3^	Prism, grey
*Z* = 8	0.07 × 0.06 × 0.02 mm

### Data collection

**Table:**

Enraf–Nonius KappaCCD diffractometer	12062 independent reflections
Radiation source: 0.2 x 2mm^2^ focus rotating anode	11048 reflections with *I* > 2σ(*I*)
Incoatec Helios focusing multilayer optics monochromator	*R*_int_ = 0.050
Detector resolution: 18.02 pixels mm^-1^	θ_max_ = 33.1°, θ_min_ = 2.9°
φ and ω scans	*h* = −43→43
Absorption correction: gaussian (*SADABS*; Bruker, 2006)	*k* = −9→9
*T*_min_ = 0.904, *T*_max_ = 0.974	*l* = −35→34
26717 measured reflections	

### Refinement

**Table:**

Refinement on *F*^2^	Secondary atom site location: difference Fourier map
Least-squares matrix: full	Hydrogen site location: inferred from neighbouring sites
*R*[*F*^2^ > 2σ(*F*^2^)] = 0.070	H-atom parameters constrained
*wR*(*F*^2^) = 0.182	*w* = 1/[σ^2^(*F*_o_^2^) + 45.2966*P*] where *P* = (*F*_o_^2^ + 2*F*_c_^2^)/3
*S* = 1.25	(Δ/σ)_max_ < 0.001
12062 reflections	Δρ_max_ = 1.82 e Å^−^^3^
510 parameters	Δρ_min_ = −1.65 e Å^−^^3^
8 restraints	Absolute structure: Parsons & Flack (2004), 5103 Friedel pairs
Primary atom site location: structure-invariant direct methods	Absolute structure parameter: 0.10 (2)

### Special details

**Table:**

Geometry. All e.s.d.'s (except the e.s.d. in the dihedral angle between two l.s. planes) are estimated using the full covariance matrix. The cell e.s.d.'s are taken into account individually in the estimation of e.s.d.'s in distances, angles and torsion angles; correlations between e.s.d.'s in cell parameters are only used when they are defined by crystal symmetry. An approximate (isotropic) treatment of cell e.s.d.'s is used for estimating e.s.d.'s involving l.s. planes.
Refinement. Refined as a 2-component inversion twin.

### Fractional atomic coordinates and isotropic or equivalent isotropic displacement parameters (Å^2^)

**Table:**

	*x*	*y*	*z*	*U*_iso_*/*U*_eq_	
Cu1	0.60730 (3)	0.75260 (14)	0.60326 (4)	0.01256 (14)	
Cu2	0.38464 (3)	−0.00703 (14)	0.28880 (4)	0.01287 (15)	
C1	0.6659 (2)	1.0247 (11)	0.7213 (3)	0.0136 (11)	
C2	0.6982 (3)	1.2023 (12)	0.7533 (4)	0.0185 (14)	
C3	0.7240 (3)	1.2116 (14)	0.8196 (4)	0.0229 (16)	
H3	0.7456	1.3318	0.8393	0.027*	
C4	0.7188 (3)	1.0482 (14)	0.8576 (4)	0.0237 (16)	
H4	0.7372	1.0550	0.9030	0.028*	
C5	0.6867 (3)	0.8753 (14)	0.8294 (3)	0.0208 (14)	
H5	0.6823	0.7651	0.8556	0.025*	
C6	0.6601 (2)	0.8606 (12)	0.7615 (3)	0.0156 (12)	
C7	0.6284 (3)	0.6718 (12)	0.7375 (3)	0.0157 (12)	
H7	0.6249	0.5783	0.7684	0.019*	
C8	0.5699 (3)	0.4285 (12)	0.6616 (4)	0.0178 (12)	
H8A	0.5804	0.3304	0.6991	0.021*	
H8B	0.5723	0.3483	0.6256	0.021*	
C9	0.5138 (3)	0.5056 (13)	0.6422 (4)	0.0206 (13)	
H9A	0.4899	0.4049	0.6097	0.025*	
H9B	0.5059	0.4997	0.6804	0.025*	
C10	0.5038 (3)	0.7342 (13)	0.6150 (4)	0.0185 (13)	
H10A	0.4662	0.7642	0.5977	0.022*	
H10B	0.5217	0.8379	0.6503	0.022*	
C11	0.5012 (3)	0.9798 (13)	0.5322 (4)	0.0229 (15)	
H11A	0.5136	1.0962	0.5648	0.027*	
H11B	0.4630	0.9777	0.5142	0.027*	
C12	0.5178 (3)	1.0307 (13)	0.4787 (4)	0.0233 (15)	
H12A	0.4982	1.1583	0.4548	0.028*	
H12B	0.5083	0.9072	0.4485	0.028*	
C13	0.5753 (3)	1.0757 (12)	0.5011 (4)	0.0200 (14)	
H13A	0.5815	1.1339	0.4647	0.024*	
H13B	0.5862	1.1867	0.5354	0.024*	
C14	0.6344 (3)	0.8169 (12)	0.4967 (3)	0.0185 (13)	
H14	0.6335	0.9055	0.4624	0.022*	
C15	0.6668 (3)	0.6281 (13)	0.5102 (3)	0.0164 (12)	
C16	0.6939 (3)	0.5967 (16)	0.4708 (3)	0.0237 (16)	
H16	0.6918	0.7040	0.4402	0.028*	
C17	0.7225 (3)	0.4177 (17)	0.4763 (4)	0.0278 (19)	
H17	0.7401	0.3996	0.4493	0.033*	
C18	0.7265 (3)	0.2608 (15)	0.5208 (4)	0.0247 (16)	
H18	0.7463	0.1346	0.5238	0.030*	
C19	0.7018 (3)	0.2845 (12)	0.5615 (4)	0.0177 (13)	
C20	0.6714 (2)	0.4730 (11)	0.5577 (3)	0.0138 (11)	
C21	0.3889 (2)	0.2648 (11)	0.3996 (3)	0.0133 (11)	
C22	0.4043 (3)	0.4517 (12)	0.4402 (3)	0.0166 (12)	
C23	0.3904 (3)	0.4786 (14)	0.4908 (4)	0.0211 (14)	
H23	0.4004	0.6064	0.5160	0.025*	
C24	0.3619 (3)	0.3216 (15)	0.5052 (4)	0.0236 (15)	
H24	0.3533	0.3407	0.5407	0.028*	
C25	0.3462 (3)	0.1381 (13)	0.4678 (4)	0.0204 (14)	
H25	0.3267	0.0306	0.4775	0.024*	
C26	0.3591 (2)	0.1099 (12)	0.4149 (4)	0.0151 (12)	
C27	0.3376 (3)	−0.0816 (12)	0.3766 (4)	0.0172 (12)	
H27	0.3175	−0.1721	0.3907	0.021*	
C28	0.3169 (2)	−0.3457 (12)	0.2958 (4)	0.0190 (13)	
H28A	0.3015	−0.4136	0.3228	0.023*	
H28B	0.3429	−0.4468	0.2933	0.023*	
C29	0.2746 (3)	−0.3076 (13)	0.2284 (5)	0.0253 (17)	
H29A	0.2504	−0.1968	0.2309	0.030*	
H29B	0.2548	−0.4433	0.2129	0.030*	
C30	0.2949 (3)	−0.2340 (14)	0.1794 (4)	0.0264 (17)	
H30A	0.3200	−0.3427	0.1781	0.032*	
H30B	0.2657	−0.2303	0.1364	0.032*	
C31	0.3389 (3)	0.0262 (13)	0.1427 (4)	0.0223 (15)	
H31A	0.3102	0.0008	0.1000	0.027*	
H31B	0.3669	−0.0770	0.1476	0.027*	
C32	0.3589 (4)	0.2539 (14)	0.1445 (4)	0.0280 (17)	
H32A	0.3293	0.3554	0.1305	0.034*	
H32B	0.3746	0.2646	0.1133	0.034*	
C33	0.3994 (3)	0.3251 (12)	0.2112 (4)	0.0178 (13)	
H33A	0.4254	0.4203	0.2060	0.021*	
H33B	0.3822	0.4078	0.2337	0.021*	
C34	0.4716 (3)	0.0908 (11)	0.2587 (3)	0.0145 (11)	
H34	0.4881	0.1910	0.2420	0.017*	
C35	0.5015 (2)	−0.0938 (11)	0.2915 (3)	0.0125 (11)	
C36	0.5534 (3)	−0.1017 (12)	0.2977 (3)	0.0161 (12)	
H36	0.5660	0.0148	0.2814	0.019*	
C37	0.5855 (3)	−0.2712 (13)	0.3264 (4)	0.0176 (13)	
H37	0.6199	−0.2732	0.3300	0.021*	
C38	0.5663 (3)	−0.4425 (12)	0.3503 (3)	0.0160 (12)	
H38	0.5882	−0.5620	0.3701	0.019*	
C39	0.5161 (2)	−0.4401 (10)	0.3453 (3)	0.0117 (10)	
C40	0.4818 (2)	−0.2639 (10)	0.3173 (3)	0.0116 (10)	
N1	0.6064 (2)	0.8803 (10)	0.5256 (3)	0.0152 (11)	
N2	0.5213 (2)	0.7704 (10)	0.5638 (3)	0.0152 (10)	
H2A	0.5066	0.6533	0.5311	0.018*	
N3	0.6041 (2)	0.6173 (10)	0.6779 (3)	0.0140 (10)	
N4	0.3424 (2)	−0.1445 (10)	0.3259 (3)	0.0147 (11)	
N5	0.3203 (2)	−0.0198 (11)	0.1927 (3)	0.0196 (12)	
H5A	0.2942	0.0922	0.1901	0.023*	
N6	0.4254 (2)	0.1346 (10)	0.2496 (3)	0.0139 (10)	
O1	0.64396 (19)	1.0198 (8)	0.6580 (2)	0.0154 (9)	
O2	0.7072 (2)	1.3649 (9)	0.7190 (3)	0.0256 (13)	
H2	0.6800	1.3917	0.6862	0.038*	
O3	0.64872 (19)	0.4898 (8)	0.5970 (2)	0.0150 (9)	
O4	0.7093 (2)	0.1270 (9)	0.6058 (3)	0.0232 (11)	
H4A	0.6839	0.1226	0.6153	0.035*	
O5	0.40226 (19)	0.2469 (8)	0.3519 (2)	0.0144 (8)	
O6	0.4346 (2)	0.6078 (9)	0.4317 (3)	0.0203 (10)	
H6	0.4406	0.5767	0.3998	0.030*	
O7	0.43578 (18)	−0.2675 (8)	0.3168 (2)	0.0147 (9)	
O8	0.5012 (2)	−0.6116 (9)	0.3707 (3)	0.0177 (10)	
H8	0.4692	−0.6048	0.3601	0.027*	

### Atomic displacement parameters (Å^2^)

**Table:**

	*U*^11^	*U*^22^	*U*^33^	*U*^12^	*U*^13^	*U*^23^
Cu1	0.0175 (3)	0.0105 (3)	0.0090 (3)	−0.0002 (3)	0.0049 (2)	0.0018 (3)
Cu2	0.0123 (3)	0.0117 (3)	0.0148 (3)	−0.0012 (3)	0.0059 (2)	0.0003 (3)
C1	0.013 (2)	0.011 (3)	0.013 (3)	0.004 (2)	0.002 (2)	−0.002 (2)
C2	0.015 (3)	0.013 (3)	0.018 (3)	0.002 (2)	−0.003 (2)	−0.004 (2)
C3	0.016 (3)	0.025 (4)	0.021 (3)	0.006 (3)	0.001 (2)	−0.008 (3)
C4	0.024 (3)	0.031 (4)	0.011 (3)	0.008 (3)	0.002 (2)	−0.005 (3)
C5	0.019 (3)	0.029 (4)	0.011 (3)	0.008 (3)	0.003 (2)	0.001 (3)
C6	0.012 (3)	0.022 (3)	0.013 (3)	0.007 (2)	0.005 (2)	0.001 (2)
C7	0.017 (3)	0.019 (3)	0.014 (3)	0.004 (2)	0.010 (2)	0.004 (2)
C8	0.024 (3)	0.018 (3)	0.014 (3)	−0.002 (2)	0.010 (2)	0.001 (2)
C9	0.022 (3)	0.021 (3)	0.022 (3)	−0.006 (3)	0.012 (3)	−0.001 (3)
C10	0.017 (3)	0.018 (3)	0.021 (3)	−0.002 (2)	0.008 (2)	−0.004 (3)
C11	0.019 (3)	0.015 (3)	0.026 (4)	0.003 (3)	0.001 (3)	0.002 (3)
C12	0.023 (3)	0.019 (4)	0.016 (3)	0.000 (3)	−0.004 (2)	0.004 (3)
C13	0.023 (3)	0.015 (3)	0.018 (3)	−0.002 (2)	0.005 (3)	0.006 (3)
C14	0.023 (3)	0.017 (3)	0.009 (3)	−0.008 (2)	0.001 (2)	0.002 (2)
C15	0.014 (3)	0.026 (3)	0.008 (3)	−0.010 (2)	0.004 (2)	−0.003 (2)
C16	0.015 (3)	0.049 (5)	0.010 (3)	−0.009 (3)	0.007 (2)	−0.003 (3)
C17	0.015 (3)	0.052 (6)	0.016 (3)	−0.008 (3)	0.007 (2)	−0.014 (3)
C18	0.015 (3)	0.034 (4)	0.026 (4)	−0.006 (3)	0.008 (3)	−0.014 (3)
C19	0.013 (3)	0.017 (3)	0.023 (3)	−0.002 (2)	0.008 (2)	−0.006 (3)
C20	0.011 (2)	0.014 (3)	0.015 (3)	−0.003 (2)	0.005 (2)	−0.003 (2)
C21	0.012 (2)	0.013 (3)	0.014 (3)	0.002 (2)	0.004 (2)	0.001 (2)
C22	0.018 (3)	0.016 (3)	0.012 (3)	−0.004 (2)	0.003 (2)	0.000 (2)
C23	0.021 (3)	0.027 (4)	0.015 (3)	−0.007 (3)	0.007 (2)	−0.006 (3)
C24	0.020 (3)	0.036 (4)	0.016 (3)	−0.005 (3)	0.009 (3)	−0.003 (3)
C25	0.018 (3)	0.025 (4)	0.020 (3)	−0.004 (3)	0.010 (2)	0.002 (3)
C26	0.007 (2)	0.018 (3)	0.022 (3)	−0.001 (2)	0.008 (2)	0.002 (2)
C27	0.013 (3)	0.016 (3)	0.024 (3)	−0.003 (2)	0.009 (2)	0.000 (3)
C28	0.009 (2)	0.018 (3)	0.034 (4)	−0.005 (2)	0.013 (2)	−0.005 (3)
C29	0.010 (3)	0.021 (3)	0.042 (5)	−0.005 (2)	0.007 (3)	−0.013 (3)
C30	0.021 (3)	0.020 (4)	0.026 (4)	0.001 (3)	−0.002 (3)	−0.008 (3)
C31	0.028 (3)	0.021 (4)	0.012 (3)	0.007 (3)	0.002 (2)	−0.001 (3)
C32	0.041 (4)	0.023 (4)	0.016 (3)	0.011 (3)	0.007 (3)	0.006 (3)
C33	0.018 (3)	0.014 (3)	0.022 (3)	0.004 (2)	0.009 (3)	0.001 (2)
C34	0.016 (3)	0.013 (3)	0.017 (3)	0.000 (2)	0.008 (2)	0.002 (2)
C35	0.010 (2)	0.016 (3)	0.015 (3)	−0.004 (2)	0.008 (2)	−0.002 (2)
C36	0.015 (3)	0.019 (3)	0.017 (3)	−0.005 (2)	0.010 (2)	−0.005 (2)
C37	0.014 (3)	0.022 (3)	0.019 (3)	−0.001 (2)	0.008 (2)	−0.004 (3)
C38	0.015 (3)	0.017 (3)	0.015 (3)	0.003 (2)	0.004 (2)	−0.002 (2)
C39	0.009 (2)	0.010 (2)	0.012 (3)	−0.0016 (18)	0.0006 (19)	−0.002 (2)
C40	0.014 (2)	0.009 (2)	0.010 (2)	0.001 (2)	0.0035 (19)	−0.001 (2)
N1	0.018 (2)	0.015 (3)	0.011 (2)	−0.004 (2)	0.0035 (19)	0.003 (2)
N2	0.018 (2)	0.011 (2)	0.014 (2)	−0.0024 (19)	0.0043 (19)	−0.001 (2)
N3	0.014 (2)	0.017 (3)	0.011 (2)	0.001 (2)	0.0052 (19)	0.005 (2)
N4	0.010 (2)	0.015 (3)	0.021 (3)	−0.0021 (19)	0.009 (2)	−0.001 (2)
N5	0.017 (2)	0.015 (3)	0.017 (3)	0.006 (2)	−0.002 (2)	−0.004 (2)
N6	0.014 (2)	0.014 (3)	0.013 (2)	0.0000 (19)	0.0049 (19)	0.002 (2)
O1	0.022 (2)	0.010 (2)	0.0090 (19)	−0.0003 (17)	0.0011 (16)	0.0011 (16)
O2	0.025 (3)	0.012 (2)	0.022 (3)	−0.001 (2)	−0.007 (2)	0.000 (2)
O3	0.020 (2)	0.012 (2)	0.015 (2)	−0.0001 (18)	0.0093 (17)	0.0026 (17)
O4	0.021 (2)	0.015 (2)	0.036 (3)	0.0031 (19)	0.014 (2)	0.001 (2)
O5	0.019 (2)	0.013 (2)	0.013 (2)	−0.0039 (17)	0.0079 (16)	−0.0012 (17)
O6	0.025 (2)	0.020 (3)	0.017 (2)	−0.009 (2)	0.009 (2)	−0.004 (2)
O7	0.0134 (19)	0.013 (2)	0.018 (2)	−0.0013 (16)	0.0073 (16)	0.0032 (18)
O8	0.015 (2)	0.014 (2)	0.021 (3)	0.0033 (17)	0.0051 (18)	0.0062 (19)

### Geometric parameters (Å, º)

**Table:**

Cu1—N1	1.939 (6)	C19—C20	1.433 (10)
Cu1—N3	1.942 (6)	C20—O3	1.314 (8)
Cu1—O3	2.049 (5)	C21—O5	1.305 (8)
Cu1—O1	2.074 (5)	C21—C26	1.417 (9)
Cu1—N2	2.228 (6)	C21—C22	1.434 (10)
Cu2—N4	1.936 (6)	C22—O6	1.362 (9)
Cu2—N6	1.948 (6)	C22—C23	1.384 (11)
Cu2—O5	2.050 (5)	C23—C24	1.392 (11)
Cu2—O7	2.084 (5)	C23—H23	0.9500
Cu2—N5	2.198 (6)	C24—C25	1.379 (12)
C1—O1	1.316 (8)	C24—H24	0.9500
C1—C2	1.423 (10)	C25—C26	1.419 (10)
C1—C6	1.428 (10)	C25—H25	0.9500
C2—O2	1.366 (10)	C26—C27	1.449 (10)
C2—C3	1.385 (11)	C27—N4	1.286 (10)
C3—C4	1.383 (13)	C27—H27	0.9500
C3—H3	0.9500	C28—N4	1.460 (9)
C4—C5	1.379 (12)	C28—C29	1.527 (12)
C4—H4	0.9500	C28—H28A	0.9900
C5—C6	1.420 (10)	C28—H28B	0.9900
C5—H5	0.9500	C29—C30	1.533 (14)
C6—C7	1.437 (11)	C29—H29A	0.9900
C7—N3	1.290 (9)	C29—H29B	0.9900
C7—H7	0.9500	C30—N5	1.479 (11)
C8—N3	1.466 (10)	C30—H30A	0.9900
C8—C9	1.542 (11)	C30—H30B	0.9900
C8—H8A	0.9900	C31—N5	1.477 (11)
C8—H8B	0.9900	C31—C32	1.514 (13)
C9—C10	1.524 (11)	C31—H31A	0.9900
C9—H9A	0.9900	C31—H31B	0.9900
C9—H9B	0.9900	C32—C33	1.543 (11)
C10—N2	1.471 (10)	C32—H32A	0.9900
C10—H10A	0.9900	C32—H32B	0.9900
C10—H10B	0.9900	C33—N6	1.471 (9)
C11—N2	1.478 (10)	C33—H33A	0.9900
C11—C12	1.521 (13)	C33—H33B	0.9900
C11—H11A	0.9900	C34—N6	1.274 (8)
C11—H11B	0.9900	C34—C35	1.434 (10)
C12—C13	1.522 (11)	C34—H34	0.9500
C12—H12A	0.9900	C35—C36	1.425 (9)
C12—H12B	0.9900	C35—C40	1.431 (9)
C13—N1	1.464 (10)	C36—C37	1.365 (11)
C13—H13A	0.9900	C36—H36	0.9500
C13—H13B	0.9900	C37—C38	1.403 (11)
C14—N1	1.294 (10)	C37—H37	0.9500
C14—C15	1.438 (11)	C38—C39	1.386 (9)
C14—H14	0.9500	C38—H38	0.9500
C15—C20	1.415 (10)	C39—O8	1.361 (8)
C15—C16	1.428 (10)	C39—C40	1.425 (9)
C16—C17	1.349 (14)	C40—O7	1.306 (8)
C16—H16	0.9500	N2—H2A	1.0000
C17—C18	1.379 (14)	N5—H5A	1.0000
C17—H17	0.9500	O2—H2	0.8400
C18—C19	1.391 (11)	O4—H4A	0.8400
C18—H18	0.9500	O6—H6	0.8400
C19—O4	1.357 (10)	O8—H8	0.8400
			
N1—Cu1—N3	176.5 (3)	C22—C23—C24	121.1 (7)
N1—Cu1—O3	92.6 (2)	C22—C23—H23	119.5
N3—Cu1—O3	87.8 (2)	C24—C23—H23	119.5
N1—Cu1—O1	92.4 (2)	C25—C24—C23	119.7 (7)
N3—Cu1—O1	90.4 (2)	C25—C24—H24	120.1
O3—Cu1—O1	121.1 (2)	C23—C24—H24	120.1
N1—Cu1—N2	91.0 (2)	C24—C25—C26	120.0 (7)
N3—Cu1—N2	85.9 (2)	C24—C25—H25	120.0
O3—Cu1—N2	125.3 (2)	C26—C25—H25	120.0
O1—Cu1—N2	113.3 (2)	C21—C26—C25	121.7 (7)
N4—Cu2—N6	178.4 (3)	C21—C26—C27	123.2 (7)
N4—Cu2—O5	92.3 (2)	C25—C26—C27	115.1 (6)
N6—Cu2—O5	87.9 (2)	N4—C27—C26	128.1 (7)
N4—Cu2—O7	90.4 (2)	N4—C27—H27	115.9
N6—Cu2—O7	90.9 (2)	C26—C27—H27	115.9
O5—Cu2—O7	116.2 (2)	N4—C28—C29	111.8 (6)
N4—Cu2—N5	90.8 (3)	N4—C28—H28A	109.3
N6—Cu2—N5	87.7 (3)	C29—C28—H28A	109.3
O5—Cu2—N5	126.1 (2)	N4—C28—H28B	109.3
O7—Cu2—N5	117.5 (2)	C29—C28—H28B	109.3
O1—C1—C2	119.5 (7)	H28A—C28—H28B	107.9
O1—C1—C6	124.4 (6)	C28—C29—C30	114.1 (6)
C2—C1—C6	116.1 (6)	C28—C29—H29A	108.7
O2—C2—C3	117.4 (7)	C30—C29—H29A	108.7
O2—C2—C1	120.5 (6)	C28—C29—H29B	108.7
C3—C2—C1	122.0 (8)	C30—C29—H29B	108.7
C4—C3—C2	120.9 (7)	H29A—C29—H29B	107.6
C4—C3—H3	119.5	N5—C30—C29	114.7 (7)
C2—C3—H3	119.5	N5—C30—H30A	108.6
C5—C4—C3	119.8 (7)	C29—C30—H30A	108.6
C5—C4—H4	120.1	N5—C30—H30B	108.6
C3—C4—H4	120.1	C29—C30—H30B	108.6
C4—C5—C6	120.5 (8)	H30A—C30—H30B	107.6
C4—C5—H5	119.7	N5—C31—C32	114.3 (7)
C6—C5—H5	119.7	N5—C31—H31A	108.7
C5—C6—C1	120.6 (7)	C32—C31—H31A	108.7
C5—C6—C7	115.7 (7)	N5—C31—H31B	108.7
C1—C6—C7	123.7 (6)	C32—C31—H31B	108.7
N3—C7—C6	126.1 (7)	H31A—C31—H31B	107.6
N3—C7—H7	116.9	C31—C32—C33	114.1 (7)
C6—C7—H7	116.9	C31—C32—H32A	108.7
N3—C8—C9	109.0 (6)	C33—C32—H32A	108.7
N3—C8—H8A	109.9	C31—C32—H32B	108.7
C9—C8—H8A	109.9	C33—C32—H32B	108.7
N3—C8—H8B	109.9	H32A—C32—H32B	107.6
C9—C8—H8B	109.9	N6—C33—C32	110.1 (6)
H8A—C8—H8B	108.3	N6—C33—H33A	109.6
C10—C9—C8	113.4 (6)	C32—C33—H33A	109.6
C10—C9—H9A	108.9	N6—C33—H33B	109.6
C8—C9—H9A	108.9	C32—C33—H33B	109.6
C10—C9—H9B	108.9	H33A—C33—H33B	108.2
C8—C9—H9B	108.9	N6—C34—C35	126.8 (6)
H9A—C9—H9B	107.7	N6—C34—H34	116.6
N2—C10—C9	113.6 (6)	C35—C34—H34	116.6
N2—C10—H10A	108.8	C36—C35—C40	120.2 (6)
C9—C10—H10A	108.8	C36—C35—C34	116.1 (6)
N2—C10—H10B	108.8	C40—C35—C34	123.6 (6)
C9—C10—H10B	108.8	C37—C36—C35	122.1 (7)
H10A—C10—H10B	107.7	C37—C36—H36	118.9
N2—C11—C12	112.9 (7)	C35—C36—H36	118.9
N2—C11—H11A	109.0	C36—C37—C38	118.5 (6)
C12—C11—H11A	109.0	C36—C37—H37	120.8
N2—C11—H11B	109.0	C38—C37—H37	120.8
C12—C11—H11B	109.0	C39—C38—C37	121.0 (6)
H11A—C11—H11B	107.8	C39—C38—H38	119.5
C11—C12—C13	114.9 (6)	C37—C38—H38	119.5
C11—C12—H12A	108.6	O8—C39—C38	117.0 (6)
C13—C12—H12A	108.6	O8—C39—C40	120.7 (6)
C11—C12—H12B	108.6	C38—C39—C40	122.3 (6)
C13—C12—H12B	108.6	O7—C40—C39	119.2 (6)
H12A—C12—H12B	107.5	O7—C40—C35	125.1 (6)
N1—C13—C12	111.7 (6)	C39—C40—C35	115.8 (6)
N1—C13—H13A	109.3	C14—N1—C13	117.3 (6)
C12—C13—H13A	109.3	C14—N1—Cu1	125.0 (5)
N1—C13—H13B	109.3	C13—N1—Cu1	117.5 (5)
C12—C13—H13B	109.3	C10—N2—C11	109.4 (6)
H13A—C13—H13B	107.9	C10—N2—Cu1	110.6 (4)
N1—C14—C15	127.7 (7)	C11—N2—Cu1	112.1 (4)
N1—C14—H14	116.1	C10—N2—H2A	108.2
C15—C14—H14	116.1	C11—N2—H2A	108.2
C20—C15—C16	119.7 (7)	Cu1—N2—H2A	108.2
C20—C15—C14	124.1 (7)	C7—N3—C8	118.8 (6)
C16—C15—C14	116.2 (7)	C7—N3—Cu1	128.2 (5)
C17—C16—C15	121.3 (8)	C8—N3—Cu1	113.0 (5)
C17—C16—H16	119.4	C27—N4—C28	117.7 (6)
C15—C16—H16	119.4	C27—N4—Cu2	125.8 (5)
C16—C17—C18	120.3 (7)	C28—N4—Cu2	116.4 (5)
C16—C17—H17	119.8	C31—N5—C30	108.4 (6)
C18—C17—H17	119.8	C31—N5—Cu2	110.6 (4)
C17—C18—C19	121.0 (8)	C30—N5—Cu2	111.6 (5)
C17—C18—H18	119.5	C31—N5—H5A	108.7
C19—C18—H18	119.5	C30—N5—H5A	108.7
O4—C19—C18	117.3 (7)	Cu2—N5—H5A	108.7
O4—C19—C20	122.1 (7)	C34—N6—C33	119.6 (6)
C18—C19—C20	120.5 (8)	C34—N6—Cu2	127.1 (5)
O3—C20—C15	124.1 (6)	C33—N6—Cu2	113.0 (4)
O3—C20—C19	118.7 (6)	C1—O1—Cu1	124.8 (4)
C15—C20—C19	117.2 (7)	C2—O2—H2	109.5
O5—C21—C26	124.4 (6)	C20—O3—Cu1	125.0 (4)
O5—C21—C22	119.7 (6)	C19—O4—H4A	109.5
C26—C21—C22	115.9 (6)	C21—O5—Cu2	125.8 (4)
O6—C22—C23	117.1 (7)	C22—O6—H6	109.5
O6—C22—C21	121.3 (7)	C40—O7—Cu2	123.9 (4)
C23—C22—C21	121.6 (7)	C39—O8—H8	109.5
			
O1—C1—C2—O2	−1.2 (10)	N4—C28—C29—C30	67.5 (8)
C6—C1—C2—O2	178.3 (6)	C28—C29—C30—N5	−65.0 (9)
O1—C1—C2—C3	−177.2 (7)	N5—C31—C32—C33	51.3 (9)
C6—C1—C2—C3	2.4 (10)	C31—C32—C33—N6	24.4 (10)
O2—C2—C3—C4	−177.0 (7)	N6—C34—C35—C36	−177.5 (7)
C1—C2—C3—C4	−0.9 (12)	N6—C34—C35—C40	2.5 (12)
C2—C3—C4—C5	−1.3 (12)	C40—C35—C36—C37	1.8 (10)
C3—C4—C5—C6	1.8 (11)	C34—C35—C36—C37	−178.2 (7)
C4—C5—C6—C1	−0.3 (10)	C35—C36—C37—C38	0.1 (11)
C4—C5—C6—C7	179.0 (7)	C36—C37—C38—C39	−0.3 (11)
O1—C1—C6—C5	177.7 (6)	C37—C38—C39—O8	−179.1 (6)
C2—C1—C6—C5	−1.8 (9)	C37—C38—C39—C40	−1.4 (10)
O1—C1—C6—C7	−1.5 (10)	O8—C39—C40—O7	0.8 (9)
C2—C1—C6—C7	179.0 (6)	C38—C39—C40—O7	−176.9 (6)
C5—C6—C7—N3	−174.3 (7)	O8—C39—C40—C35	−179.4 (6)
C1—C6—C7—N3	4.9 (11)	C38—C39—C40—C35	3.0 (9)
N3—C8—C9—C10	26.9 (8)	C36—C35—C40—O7	176.7 (6)
C8—C9—C10—N2	50.2 (8)	C34—C35—C40—O7	−3.4 (11)
N2—C11—C12—C13	−68.3 (9)	C36—C35—C40—C39	−3.2 (9)
C11—C12—C13—N1	69.7 (9)	C34—C35—C40—C39	176.8 (6)
N1—C14—C15—C20	2.6 (11)	C15—C14—N1—C13	−178.0 (6)
N1—C14—C15—C16	−179.7 (7)	C15—C14—N1—Cu1	8.0 (10)
C20—C15—C16—C17	2.5 (10)	C12—C13—N1—C14	119.7 (7)
C14—C15—C16—C17	−175.4 (7)	C12—C13—N1—Cu1	−65.8 (7)
C15—C16—C17—C18	−0.5 (11)	C9—C10—N2—C11	169.2 (6)
C16—C17—C18—C19	−0.9 (11)	C9—C10—N2—Cu1	−66.8 (6)
C17—C18—C19—O4	−177.3 (7)	C12—C11—N2—C10	179.7 (6)
C17—C18—C19—C20	0.3 (11)	C12—C11—N2—Cu1	56.7 (7)
C16—C15—C20—O3	178.6 (6)	C6—C7—N3—C8	−174.8 (6)
C14—C15—C20—O3	−3.7 (10)	C6—C7—N3—Cu1	6.3 (10)
C16—C15—C20—C19	−2.9 (9)	C9—C8—N3—C7	96.8 (7)
C14—C15—C20—C19	174.7 (6)	C9—C8—N3—Cu1	−84.1 (6)
O4—C19—C20—O3	−2.4 (10)	C26—C27—N4—C28	179.8 (7)
C18—C19—C20—O3	−179.8 (6)	C26—C27—N4—Cu2	4.6 (11)
O4—C19—C20—C15	179.0 (6)	C29—C28—N4—C27	115.9 (8)
C18—C19—C20—C15	1.6 (10)	C29—C28—N4—Cu2	−68.4 (6)
O5—C21—C22—O6	−2.7 (10)	C32—C31—N5—C30	171.7 (6)
C26—C21—C22—O6	177.6 (6)	C32—C31—N5—Cu2	−65.7 (7)
O5—C21—C22—C23	179.1 (7)	C29—C30—N5—C31	178.6 (6)
C26—C21—C22—C23	−0.7 (10)	C29—C30—N5—Cu2	56.5 (7)
O6—C22—C23—C24	−176.5 (7)	C35—C34—N6—C33	−176.2 (7)
C21—C22—C23—C24	1.8 (12)	C35—C34—N6—Cu2	10.9 (11)
C22—C23—C24—C25	−1.5 (12)	C32—C33—N6—C34	105.9 (8)
C23—C24—C25—C26	0.1 (12)	C32—C33—N6—Cu2	−80.2 (7)
O5—C21—C26—C25	179.5 (6)	C2—C1—O1—Cu1	168.1 (5)
C22—C21—C26—C25	−0.8 (9)	C6—C1—O1—Cu1	−11.4 (9)
O5—C21—C26—C27	−3.6 (10)	C15—C20—O3—Cu1	−5.4 (9)
C22—C21—C26—C27	176.1 (6)	C19—C20—O3—Cu1	176.2 (4)
C24—C25—C26—C21	1.1 (11)	C26—C21—O5—Cu2	−0.7 (9)
C24—C25—C26—C27	−176.1 (7)	C22—C21—O5—Cu2	179.5 (5)
C21—C26—C27—N4	1.7 (12)	C39—C40—O7—Cu2	171.7 (4)
C25—C26—C27—N4	178.8 (7)	C35—C40—O7—Cu2	−8.1 (9)

### Hydrogen-bond geometry (Å, º)

**Table:**

*D*—H···*A*	*D*—H	H···*A*	*D*···*A*	*D*—H···*A*
O2—H2···O3^i^	0.84	1.95	2.699 (8)	147
O4—H4*A*···O1^ii^	0.84	1.90	2.686 (8)	156
O6—H6···O7^i^	0.84	2.08	2.758 (8)	137
O6—H6···O8^i^	0.84	2.40	3.101 (8)	142
O8—H8···O5^ii^	0.84	2.05	2.806 (7)	149
N2—H2*A*···O6	1.00	2.36	3.163 (8)	137

Symmetry codes: (i) *x*, *y*+1, *z*; (ii) *x*, *y*−1, *z*.
