# Supplementary figures and images for: Crystal structure of {6,6′-dihy­droxy-2,2′-[imino­bis­(propane-1,3-diyl­nitrilo­methanylyl­idene)]diphenolato-κ5 O 1,N,N′,N′′,O 1′}copper(II)
Source: Acta Crystallogr E Crystallogr Commun. 2015 Oct 24;71(Pt 11):m203–4. doi: 10.1107/S2056989015019684 (PMC4645064; doi:10.1107/S2056989015019684)

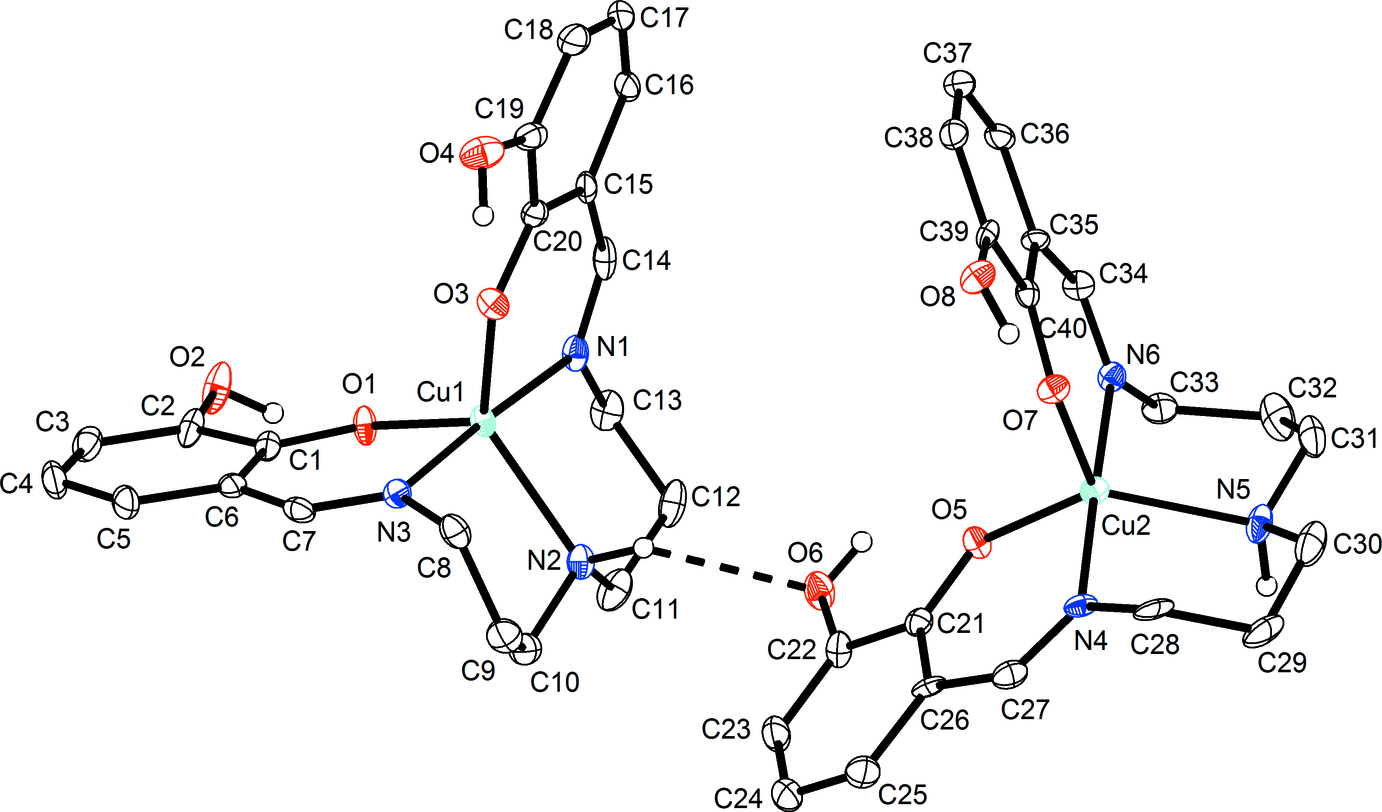

Supplement: Supplementary file 3 [file e-71-0m203-fig1.tif]

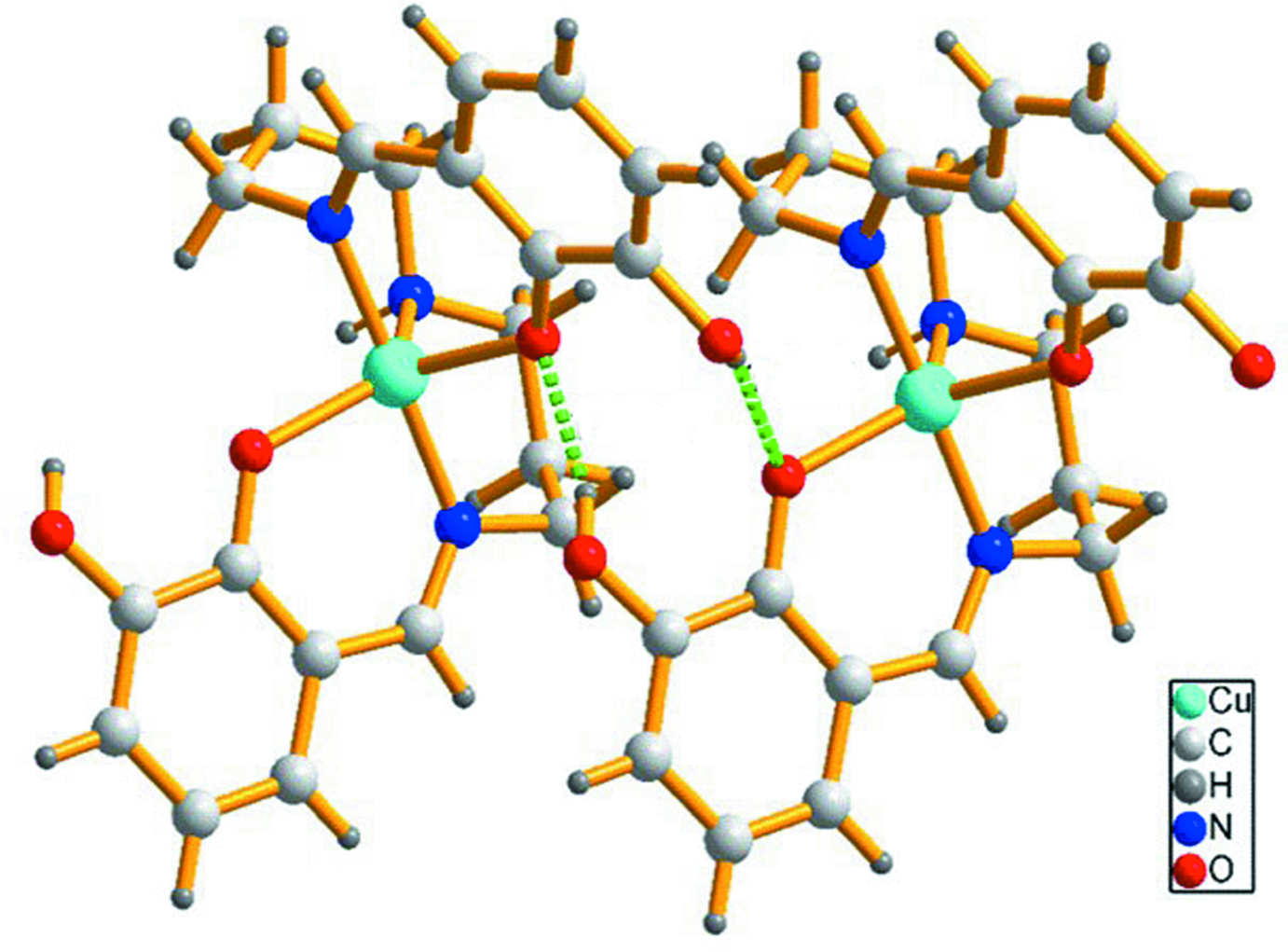

Supplement: Supplementary file 4 [file e-71-0m203-fig2.tif]
